# Why the young sleep longer

**DOI:** 10.7554/eLife.56833

**Published:** 2020-04-27

**Authors:** Budhaditya Chowdhury, Orie T Shafer

**Affiliations:** Advanced Science Research Center, The Graduate Center, The City University of New YorkNew YorkUnited States

**Keywords:** synapse, sleep, dopamine, ontogeny, central complex, brain development, *D. melanogaster*

## Abstract

A transcription factor helps young flies to sleep longer by delaying the maturation of a neural network that controls sleep.

**Related research article** Chakravarti Dilley L, Szuperak M, Gong NN, Williams CE, Saldana RL, Garbe DS, Syed MH, Jain R, Kayser MS. 2020. Identification of a molecular basis for the juvenile sleep state. *eLife* **9**:e52676. doi: 10.7554/eLife.52676

From absorbing new languages to mastering musical instruments, young children are wired to learn in ways that adults are not (Johnson and Newport, 1989). This ability coincides with periods of intense brain plasticity during which neurons can easily remodel their connections (Hubel and Wiesel, 1970). Many children are also scandalously good sleepers, typically getting several more hours of sleep per night than their parents (Jenni and Carskadon, 2007). As sleep deprivation has negative effects on learning and memory, learning like a child likely requires sleeping like one (Diekelmann and Born, 2010). Yet, how the ability to sleep for longer is synchronized with windows of high brain plasticity is not fully understood.

Sleep is deeply conserved through evolution, and examining how it develops in ‘simple organisms’ should provide fundamental insights relevant to humans. For instance, like human teenagers, one-day old *Drosophila melanogaster* flies sleep twice as much as mature adults, and disrupting the sleep of young flies has lasting effects on learning and behavior (Seugnet et al., 2011; Kayser et al., 2014). Now, in eLife, Matthew Kayser from the University of Pennsylvania and co-workers – including Leela Chakravarti Dilley as first author – report new regulatory mechanisms that promote sleep in young flies (Chakravarti Dilley et al., 2020).

The team started by searching for genes which, when knocked down, would reduce the difference in sleep duration between younger and older adult flies. A gene called *pdm3* fit the bill by reducing sleep in juveniles. This gene codes for a transcription factor that belongs to a family known to regulate normal brain development. Chakravarti Dilley et al. further determined that *pdm3* helps to establish correct sleep patterns for one-day-old flies during the pupal stage, when the relatively simple brain of a larva develops into the complex brain of the adult insect – a period of radical change that puts human puberty to shame.

Previous work on *pdm3* mutants revealed aberrations in the way dopaminergic neurons reach and connect with neurons in the central complex, a region of the brain that is known to regulate sleep. There, the dopaminergic neurons encourage wakefulness by inhibiting cells called dFSB neurons, which promote sleep (Pimentel et al., 2016). In flies, the density of connections between dopaminergic and dFSB neurons normally increases over the first few days of adult life. Chakravarti Dilley et al. therefore explored whether *pdm3* might regulate how dopaminergic neurons innervate the central complex. This revealed that when *pdm3* was knocked down, one-day-old flies already showed levels of innervation that rivaled those seen in mature adults (Figure 1).

**Figure 1.**
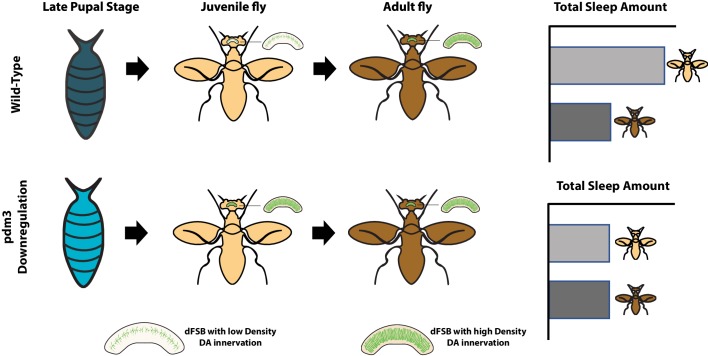
How the transcription factor pdm3 preserves high levels of sleep in young flies. Expression of *pdm3* during the pupal stage (top row) delays the innervation of the dFSB neurons (which promote sleep) by dopaminergic neurons (green) that encourage wakefulness. The progressive innervation of these neurons as the fly ages results in adult flies (dark) spending less time asleep than young flies (pale). Knock down of *pdm3* (bottom row) results in premature innervation, leading to young flies spending much less time asleep. dFSB: dorsal fan-shaped body; DA: dopaminergic.

Given that *pdm3* encodes a transcription factor, the team then searched for genes that regulate sleep and whose expression was altered by *pdm3* being knocked down. These experiments suggested that *pdm3* suppresses the expression of *msp300*, a gene from a family involved in synapse maturation. And indeed, knocking down both *pdm3* and *msp300* resulted in flies that developed normally in terms of sleep patterns and dopaminergic innervation of the central complex.

Perturbing neural development, especially during windows of high plasticity, can have a long-lasting impact on the ability for the brain to work properly (Marín, 2016). A lack of sleep could lead to such perturbations, as evidenced by the fact that disrupting sleep in early childhood or adolescence has long-term effects on behavior (Taveras et al., 2017; Roberts et al., 2009). Understanding how sleep is synchronized with periods of intense development may help to develop better therapeutic interventions that lessen long-term brain damage.
